# The Microbial Production of Polyhydroxyalkanoates from Waste Polystyrene Fragments Attained Using Oxidative Degradation

**DOI:** 10.3390/polym10090957

**Published:** 2018-08-29

**Authors:** Brian Johnston, Iza Radecka, David Hill, Emo Chiellini, Vassilka Ivanova Ilieva, Wanda Sikorska, Marta Musioł, Magdalena Zięba, Adam A. Marek, Daniel Keddie, Barbara Mendrek, Surila Darbar, Grazyna Adamus, Marek Kowalczuk

**Affiliations:** 1 Wolverhampton School of Biology, Chemistry and Forensic Science, Faculty of Science and Engineering, University of Wolverhampton, Wolverhampton WV1 1LY, UK; B.Johnston@wlv.ac.uk (B.J.); D.Hill@wlv.ac.uk (D.H.); D.Keddie@wlv.ac.uk (D.K.); Barbara.Mendrek@wlv.ac.uk (B.M.); S.Darbar@wlv.ac.uk (S.D.); 2 Laboratorio Materiali Polimerici Ecocompatibili (LMPE), via Nuova, 44/a, Segromigno in Monte, 55018 Lucca, Italy; emo.chiellini@lmpe.eu (E.C.); vassilka.ilieva@lmpe.eu (V.I.I.); 3 Centre of Polymer and Carbon Materials, Polish Academy of Sciences, 41-800 Zabrze, Poland; w.sikorska@cmpw-pan.edu.pl (W.S.); mmusiol@cmpw-pan.edu.pl (M.M.); mzieba@cmpw-pan.edu.pl (M.Z.); grazyna.adamus@cmpw-pan.edu.pl (G.A.); 4 Department of Chemical Organic Technology and Petrochemistry, Silesian University of Technology, 44-100 Gliwice, Poland; adam.a.marek@polsl.pl

**Keywords:** polyhydroxyalkanoates (PHAs), polystyrene (PS), prodegraded, *Cupriavidus necator*, fermentation, mass spectrometry, bioplastics, recycling

## Abstract

Excessive levels of plastic waste in our oceans and landfills indicate that there is an abundance of potential carbon sources with huge economic value being neglected. These waste plastics, through biological fermentation, could offer alternatives to traditional petrol-based plastics. Polyhydroxyalkanoates (PHAs) are a group of plastics produced by some strains of bacteria that could be part of a new generation of polyester materials that are biodegradable, biocompatible, and, most importantly, non-toxic if discarded. This study introduces the use of prodegraded high impact and general polystyrene (PS0). Polystyrene is commonly used in disposable cutlery, CD cases, trays, and packaging. Despite these applications, some forms of polystyrene PS remain financially and environmentally expensive to send to landfills. The prodegraded PS0 waste plastics used were broken down at varied high temperatures while exposed to ozone. These variables produced PS flakes (PS1–3) and a powder (PS4) with individual acid numbers. Consequently, after fermentation, different PHAs and amounts of biomass were produced. The bacterial strain, *Cupriavidus necator* H16, was selected for this study due to its well-documented genetic profile, stability, robustness, and ability to produce PHAs at relatively low temperatures. The accumulation of PHAs varied from 39% for prodegraded PS0 in nitrogen rich media to 48% (*w*/*w*) of dry biomass with the treated PS. The polymers extracted from biomass were analyzed using nuclear magnetic resonance (NMR) and electrospray ionization tandem mass spectrometry (ESI-MS/MS) to assess their molecular structure and properties. In conclusion, the PS0–3 specimens were shown to be the most promising carbon sources for PHA biosynthesis; with 3-hydroxybutyrate and up to 12 mol % of 3-hydroxyvalerate and 3-hydroxyhexanoate co-monomeric units generated.

## 1. Introduction

**Figure 1:**
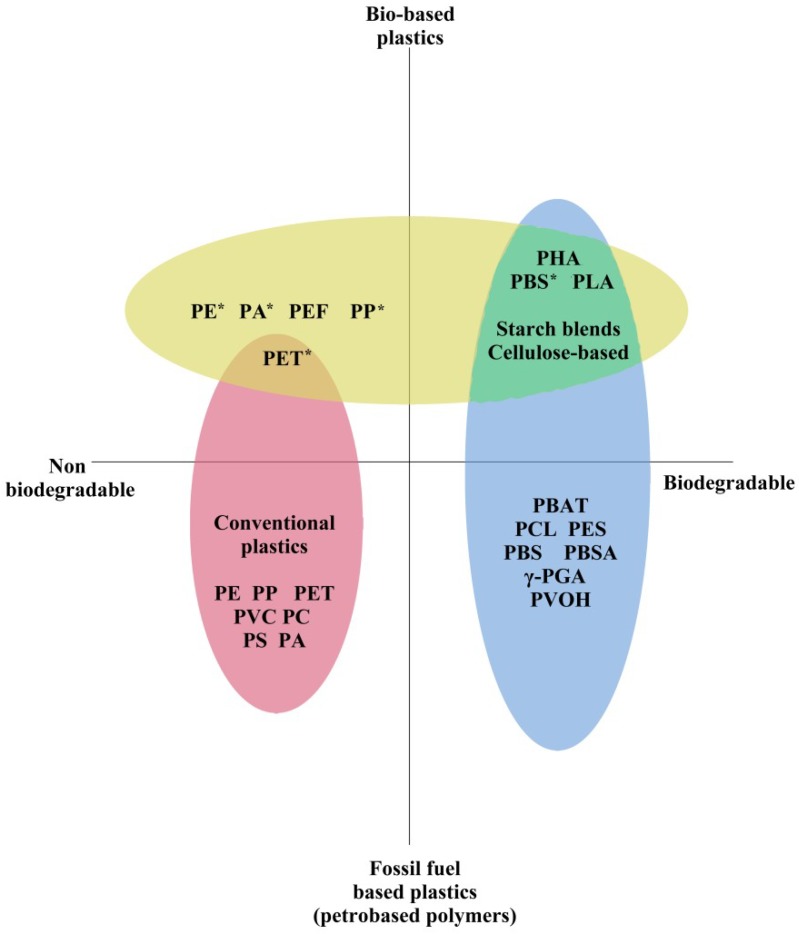
Plastics and bio-based polymers’ diversity and comparison diagram. Abbreviations include: PA—Polyamide, PBAT—Polybutyrate Adipate Terephthalate, PCL—Polycaprolactone, PE—Polyethylene, PEF—Polyethylene Furanoate, PES—Polyether Sulphone, Polyethylene Terephthalate—PET, PP—Polypropylene, PC—Polycarbonate, PHA—Polyhydroxyalkanoate, γ-PGA—Poly(Gamma Glutamic Acid), PLA—Poly(Lactic Acid), PS—Polystyrene, PBS—Poly(Butylene Succinate), PBSA—Poly(Butylene Succinate Adipate), PVC—Polyvinyl chloride, PVOH—Poly(vinyl alcohol), adapted from [13]. The * indicates polymers that have bio-based origins plus fossil fuel synthesis routes.

The “traditional” plastics derived from coal, natural gas, and oil are one of the most environmentally harmful substances produced by humans, but they are also an important and useful part of our society [1]. From mobile phones to packaging, it is difficult to imagine a world without them. Plastics are extremely versatile; they can be transparent, cost-effective, lightweight, strong, and durable, and they also possess vital properties that make them useful in medical, agricultural, domestic, and industrial applications [2]. While these properties are desirable, the highly stable structure of plastics also makes the process of natural degradation difficult; therefore, these materials are overused, and they persist in soil, marine, and freshwater ecosystems when they are irresponsibly dumped [3,4]. Even attempting to destroy plastics via incineration can cause major air pollution by releasing toxins, like dioxins, furans, mercury, and polychlorinated biphenyls [1]. Polyhydroxyalkanoates (PHAs) are a family of polyhydroxyesters with 3-, 4-, 5- and 6-hydroxyalkanoic acids that are biocompatible, biodegradable, and non-toxic organic polyesters synthesized by certain bacteria and plants from renewable sources [5,6]. PHAs function as a storage reservoir of carbon that can be accessed whenever required by the microorganism, often in the form of intercellular granules [6]. Currently, PHAs are being used either as a blend or in its pure form in a variety of applications; scl-PHAs (short chain length) are predominantly used to produce food packaging and disposable items, and mcl-PHAs (medium chain length-characterized as elastomers) are appropriate for high value-added applications, such as surgical stitching, implants, and drug delivery systems [5,7,8]. There is also the opportunity to functionalize PHB chemically and/or enzymatically (as discussed later), which is among one of the most impervious polymers to be processed in certain plastic items [9]. Global use and industrialization of PHAs is limited due to high production costs, which results in higher prices than petrol-based polymers, such as polypropylene (PP) or polyethylene (PE), that have a price of approximately US $0.60–0.87/lb [10,11]. PHAs, on the other hand, have costs up to four times greater, between US $2.25–2.75/lb [10,11]. Some businesses have industrialized the production of PHAs, but problems with the purity of substrates (like glucose), the issue that some carbon sources take from human food supplies, and the large amounts of solvents required for the extraction stages makes large scale synthesis difficult [12]. There are a wide range of microbes capable of producing intracellular or extracellular depolymerise enzymes for PHAs, which means it is readily degradable and environmentally safe, as opposed to many other plastics, including full carbon backbone mass polymers, like polystyrene (PS), PP, PE, and others (Figure 1) [6,13]. Besides those polymers featured in Figure 1, there is a growing market of bio-based building blocks used as precursors for bioplastic production, and of these, succinic acid, 1,4-butanediol, and monoethylene glycol are the most promising [14].

PS (C_8_H_8_)*_n_* is fabricated by polymerizing styrene, a building-block chemical used in the manufacture of many products [15]. It is an extremely versatile plastic that is structurally stable and is often used in products, such as CD cases, and drinks and food packaging. Small amounts of styrene can remain in PS after manufacture; however, the Food and Drug Administration (FDA) has evaluated both the safety of PS and the migrating styrene molecules and found that they are both safe for use. When PS is combined with additives, colorants, or other plastics, it can also be used to make electrical devices, plant pots, or even toys [15]. When PS is made into a foam material, expanded PS (EPS), or extruded PS (XPS), it can be used for cushions or insulation [15]. PS has a resin identification code of 6, therefore, it is recyclable, although its biodegradation can take hundreds of years and it is also relatively resistant to photo-oxidation [16]. As mentioned, PS is widely used, and now there is an abundance of it in outdoor environments. This includes coastal areas, especially in its foam form, and increasingly in the oceans as styrene monomers (SM), styrene dimers (SD), and styrene trimers (ST). PS poses a significant hazard to marine life in its high molar mass form through entanglement and smothering, and in its low molar mass form, potentially through bioaccumulation [16]. Consequently, PS appears in the red non-biodegradable section of Figure 1 with other fossil fuel based plastics, meaning PS production leaves a large carbon footprint. Moreover, due to the short lifespan of packaging, a large proportion of PS ends up in landfills, and approximately 13–20% of the solid waste produced is composed of plastic [17]. Although some European Union (EU) members have banned using landfills, approximately 50% of plastic waste still ends up in one [18]. In developing countries, plastic consumption and pollution (often marine) is more serious, and it is also less regulated; plastic usage there is believed to be greater than the world average due to more rapid rates of economic growth and urbanization [18,19,20].

**Figure 2:**
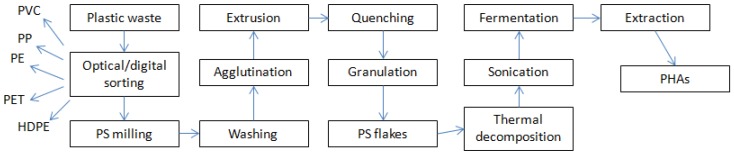
Proposed recycling stages, adapted from [22].

Life Cycle Assessment (LCA) studies have shown that the mechanical recycling of plastic materials is more favorable than other management procedures in relation to overall energy use and emissions contributing to global warming [21]. The potential pathway of this study’s overall process is summarized in Figure 2. The quality of waste plastic materials for use in processes such as this one relies on the degree of degradation, the degree of mixing (whether there are impurities or not), and the presence of low molecular weight compounds (additives, contaminants, and degradation products) [22,23,24].

The mechanical recycling of styrene polymers, such as high impact polystyrene (HIPS), has increased over the last decade due to the European Directives on Waste of Electric and Electronic Equipment (WEEE) and End-of-Life Vehicles (ELV) as styrenic plastics are used in their manufacture [23]. HIPS are a multiphase system with a PS rigid matrix and dispersed rubber particles (polybutadiene) that enhances the blend’s impact properties [23]. For environmental, social, and economic reasons, innovative and diverse strategies need to be engaged with to encourage the use of alternative biobased plastics that can equal, or even surpass, conventional mass plastics. Waste general purpose PS (GPPS) is a carbon source that could be used in this context to provide an avenue for value-added biopolymer synthesis through fermentation. The utilization of waste PS in this manner would also be in line with the recent global effort to transform Europe’s (and eventually the world’s) economy into a more sustainable one [25]. In fact, to implement the Circular Economy Action Plan, in January 2018, the European Commission endorsed a new set of measures that includes plans to modify the way plastics are designed, manufactured, applied, and recycled. They also intend that by 2030, all plastic packaging should be recyclable [25].

We propose that the fermentation of prodegraded PS (Figure 2) could be one application of a fraction of global plastic solid waste (PSW) using oxidative degradation as promoted by specific microbial strains [26]. The prodegraded PS had an acid number of 22 (see Section 3.1), indicating the presence of carboxylic groups in addition to ketone, acid, or aldehyde C=O groups [5,27]. From previous studies, the hydrophilicity of samples can be increased using thermal treatment to increase the acid number, followed by ultrasound sonication to reduce the average molecular mass to stimulate bacterial metabolism [5]. For alkane metabolism (aerobic), the most common pathway is the oxidation of the terminal methyl group into a carboxylic acid through an alcohol intermediate, which is succeeded by complete mineralization via the β-oxidation pathway [5,28,29]. The degradation of styrene has been reported previously in *Pseudomonas putida* CA-3, where epoxidation of styrene occurs, followed by isomerization of the epoxide to form phenylacetaldehyde, which is then oxidized to phenylacetic acid and converted to phenylacetylcoenzyme A (CoA) before further oxidization to acetyl-CoA [30]. The novelty of this study is that prodegraded PS is the carbon source used, and NMR has been tasked to analyze the thermal degradation process in detail, and, therefore, show the viability of PS as a carbon source with fermentation analysis and polymer sequence characterization. The bacterial strain, *Cupriavidus necator* H16 (formerly *Ralstonia eutropha*), was selected for this study as it is a reliable producer of PHAs, grows well at relatively low temperatures, has limited immunity to some heavy metals, and has a stable and well-documented genetic profile [5,31,32,33]. After the PHAs are generated, they are stored as granules, sometimes referred to as “carbonosomes” [31,32,33,34]. These microbes are known to effectively synthesize PHAs (up to 85% PHA per dry cell weight) under famine and environmentally harsh conditions using a wide range of carbon sources, including: Fatty acids, crude glycerol, hemicellulose, liquefied wood, and methane gas [34,35,36,37,38]. The removal of PHAs from biomass uses lyophilization and hot solvent extraction, followed by precipitation in hexane [5,33,35].

In summary, this study details the molecular level structural analysis of PHAs synthesized using *C. necator* as fed by prodegraded PS (PS0) waste and pretreated samples with thermal oxidation processes (PS1 to 4), as additional carbon sources in nitrogen rich tryptone soya broth (TSB) growth media and nitrogen limited, basal salts media (BSM) during optimal fermentation periods.

## 2. Materials and Methods

### 2.1. Carbon Source

The prodegraded PS flakes were generously provided by Laboratorio Materiali Polimerici Ecocompatibili (LMPE, Capannori, Italy). It was a blend of 60% GPPS and 25% of HIPS loaded with 15% of a GPPS master batch containing 5% of Mn-myristicate and 5% of Co-stearate as a pro-oxidant/pro-degradant additive.

The flakes were used as received and classified as PS0. They were then subjected to thermal oxidation and these prodegraded samples were designated PS1–4. This treatment was undertaken at the Department of Chemical Organic Technology and Petrochemistry, Silesian University of Technology Gliwice, Poland. The PS0 flakes (3 g) were placed in a 100 mL glass-reactor and immersed in a thermal bath. The process was carried out in a two-phase system: Gas-solid phase. Oxygen-ozone mixture (75 mg O_3_/L) was introduced into the reactor with a flow rate of 7.5 L/h and the duration of the processes was 20 h. The basic property of the oxidized products was the acid number (AN), which was determined according to PN-EN ISO 2114:2000 standard “Plastics (polyester resins) and paints and varnishes (binders)—Determination of partial acid value and total acid value”. The PS0–4 structural details can be found in Section 3.1.

### 2.2. Microorganism

The bacterial strain selected for the biosynthesis of PHAs with O-PS and PS flakes/powders in TSB or BSM media was *Cupriavidus necator* H16 (NCIMB 10442, ATCC 17699) obtained from NCIMB, Aberdeen, United Kingdom (freeze-dried and kept at a temperature of −20 °C). Before the study began, freeze-dried cultures were revived and grown overnight at 30 °C (their optimum) in nitrogen rich TSB at 150 rpm. The bacterial strain was then sub-cultured on tryptone soya agar (TSA) plates and incubated for 24 h at 30 °C.

### 2.3. Growth Media and Chemicals

The TSB and TSA for bacterial growth were purchased from Lab M Ltd. (Lancashire, UK). Both types of growth media were prepared under aseptic conditions, using standard procedures. The BSM used consisted of distilled water, 1 g/L K_2_HPO_4_, 1 g/L KH_2_PO_4_, 1 g/L KNO_3_, 1 g/L (NH_4_)_2_SO_4_, 0.1 g/L MgSO_4_·7H_2_O, 0.1 g/L NaCl, and 10 mL/L of trace element solution. The trace element solution contained: 2 mg/L CaCl_2_, 2 mg/L CuSO4·5H2O, 2 mg/L MnSO_4_·5H_2_O, 2 mg/L ZnSO_4_·5H_2_O, 2 mg/L FeSO_4_, and 2 mg/L (NH_4_)_6_Mo_7_O_24_·4H_2_O. BSM salts were purchased from BDH Chemicals Ltd. (Poole, UK). The Ringer’s solution was purchased from Lab M Ltd. (Lancashire, UK) and a quarter strength tablet was dissolved in 500 mL of deionized water. All media were sterilized in an autoclave (Priorclave Ltd, London, UK) at standard conditions (121 °C for 15 min).

### 2.4. Fermentation Procedure

Bacterial starter cultures were prepared using 20 mL TSB (in 50 mL flasks). These flasks were inoculated with a single *C. necator* colony from a TSA spread plate. Those cultures were then incubated (aerobically) for 24 h at 30 °C at 150 rpm in a rotary incubator (Incu-Shake MIDI, Shropshire, UK). After 24 h, these cultures were tested for contamination by standard Gram staining and microscope techniques. Shake flask culture analysis was done in triplicate using 500 mL wide neck conical flasks. Each flask used 1 g of PS or the oxidized PS1–4 flakes and powder that was first put into a 100 mL beaker and sonicated (in 50 mL of the appropriate media) for 8 min at 0.5 active and passive intervals with a power of 70% using a Bandelin Electronic sonicator, (Berlin, Germany) to form an emulsion of either TSB/PS/O-PS or BSM/PS/O-PS. This emulsion was sampled for sterility by spread plating. The sterile emulsion was then added to 200 mL of sterile TSB or BSM in a 500 mL flask, followed by 1000 µL of the starter culture, giving a total volume of 250 mL and an equal concentration of bacteria for comparison. The experimental control was 200 mL TSB or BSM, inoculated with 1000 µL of starter culture with no PS/O-PS flakes or powder. All flasks were incubated in a rotary incubator under the standard growth conditions for *C. necator* stated for 48 h.

The viable cell counts were undertaken using the techniques of Miles and Misra [39]. In summary, 5 mL samples were aseptically collected from growing cultures at 0, 3, 24, 27, and 48 h. Each sample was serially diluted from 10^−1^ to 10^−9^ and then 20 µL of each dilution was pipetted onto sectioned TSA plates in triplicate. These plates were then incubated at 30 °C for 48 h after which the colonies were counted and shown in log10 CFU mL^−1^ (Section 3.5).

### 2.5. PHA Extraction Procedure

The extraction of PHAs was performed after 48 h fermentation periods. The flasks were removed from their incubators and the contents were filtered with a sieve to remove any PS flakes or powder residue. The filtrate was then separated into tubes and centrifuged in a Sigma 6-16KS centrifuge (Sigma Laborzentrifugen GmbH, Osterode am Harz, Germany) for 10 min at 4500 rpm at room temperature. At this point the supernatant was discarded and the pellet containing the biomass was frozen overnight at −20 °C. The biomass was then lyophilized in an Edwards freeze-drier (Modulyo, Crawley, UK) for 48 h at −40 °C and 5 MBAR. The dry biomass was then weighed and recorded as the cell dry weight (CDW). For extraction, the sample was placed inside an extraction thimble capped with cotton and, using Soxhlet extraction, for 48 h with HPLC grade chloroform, and the PHA was collected as a chloroform/biopolymer mixture. This stage was followed by rotation evaporation (at 47–50 °C) to remove the chloroform. Polymer precipitation using *n*-hexane was performed in a 250 mL round-bottom flask and then this was separated further by filtration (using Watman No. 1 paper, Watman Laboratory, Maidstone, Kent, UK). The PHA sample was then left in a sterile fume cupboard to dry before the yield was recorded using:

PHA percentage yield = (weight of extracted biopolymer)/(dry cell biomass weight) × 100.

### 2.6. Characterization Techniques

#### 2.6.1. GPC

Gel permeation chromatography (GPC) was performed to determine the molar mass and molecular mass distribution of the biopolymers produced and the PS flakes and powder used throughout the investigation. A TOSOH EcoSec HLC/GPC 8320 (Tosoh Bioscience, Yamaguchi, Japan) system was used, equipped with an RI and UV detector (Tosoh Bioscience, Yamaguchi, Japan), operating at a temperature of 40 °C. The column used was TSKgel HZM-N (Tosoh Bioscience, Yamaguchi, Japan), calibrated against PS standards. The UV detector was set at a wavelength of 254 nm. Chloroform (HPLC grade) was used as the eluent, at a flow rate of 0.25 mL/min. A sample volume of 3 µL was injected into the system using an autosampler. The PS0–4 GPC analysis included: (i) PSx pre-sonication; (ii) post-sonication; and (iii) post-fermentation. The number-average molar mass (*M*_n_) and the dispersity (*M*_w_/*M*_n_) of the PS0–4 as well as the obtained PHA was determined. Those samples in chloroform were passed through membrane filters with nominal pore sizes of 0.2 μm (PTFE, Fisherbrand, Shanghai, China). The results were evaluated using EcoSEC Analysis software from Tosoh Bioscience, Yamaguchi, Japan.

#### 2.6.2. FTIR

The absorbance spectra were recorded by Fourier-transform infrared spectroscopy (FTIR) using a DuraScope (Genesis II) spectrophotometer (Smiths Detection Inc., Danbury, CT, USA) at an accumulation setting of 64 scans, with a JASCO ATR attachment. The samples were analyzed as received. The PS0, oxidized PS1–4, and PHAs (donated as PSx-PHA, where x means the number of the oxidized PS sample) samples were analyzed and their FTIR spectra were compared.

#### 2.6.3. NMR

The proton nuclear magnetic resonance (^1^H-NMR) spectra for this study were recorded with a Bruker Avance II (Bruker, Rheinstetten, Germany). The system operated at 600 MHz, with 64 scans, 2.65 s acquisition time, and an 11 µs pulse width. ^13^C-NMR spectra were recorded with a Bruker Avance II operating at 150.9 MHz, with 20,480 scans, 0.9088 s acquisition times, and 9.40 s pulse width. The ^1^H-NMR and ^13^C-NMR spectra were run in CDCl_3_ (room temperature), using tetramethylsilane (TMS) as an internal standard. Homonuclear correlation spectroscopy (COSY), Heteronuclear Multiple Bond Correlation (HMBC), and Heteronuclear Multiple Quantum Coherence (HMQC) were also performed on the same device.

The NMR spectra for analysis of the carbon sources (PS0–4) were recorded on a 400 MHz JEOL NMR spectrometer JNM-ECZ400R/M1 (Akishima, Tokyo, Japan). Samples consisted of polystyrene (PS0–4; 10–20 mg) dissolved in CDCl_3_. All spectra were internally referenced to the residual solvent [40]. ^1^H-NMR spectra were acquired with a 45° pulse and an inter-pulse delay of 5 s across 16 transients, with an acquisition time of 2.18628 s and a pulse width of 6.48 µs. Proton-decoupled ^13^C-NMR spectra were recorded with a 30° pulse and inter pulse delay of 5 s across 4097 transients, with an acquisition time of 1.03809 s and a pulse width of 10.338 µs. Phased Heteronuclear Single Quantum (HSQC) spectra were recorded using a matrix consisting of 256 × 819 points across eight scans, with a relaxation delay of 3 s. Heteronuclear Multiple Bond Correlation (HMBC) spectra were recorded using a matrix consisting of 512 × 1638 points across eight scans, with a relaxation delay of 3 s. Homonuclear Correlation Spectroscopy (COSY) spectra were recorded by using a matrix of 1024 × 1024 points across 1 scan, with a relaxation delay of 3 s.

#### 2.6.4. TGA

The thermal characterization was done using a Mettler-Toledo TGA/DSC STARe System (TA Instruments, New Castle, DE, USA). A heating rate of 10 °C·min^−1^ was used from 25 to 800 °C under a constant flow of 60 mL/min of nitrogen. The TGA data were analyzed using the Mettler-Toledo Star System SW 9.30. TG thermograms were received for all investigated samples (PS0, the oxidized PS1–4, and PHAs produced during fermentations).

#### 2.6.5. ESI-MS/MS

Electrospray ionization mass spectrometry (ESI-MS) analysis was conducted with a Finnigan LCQ ion trap mass spectrometer (Thermo Finnigan LCQ Fleet, San Jose, CA, USA). The oligomer samples were prepared as described by Johnston et al. [5] according to the mechanism presented in Kawalec et al. [41]. Specimens were dissolved in a water/methanol system (1:1 *v*/*v*), and the solutions were introduced into the ESI source (at a rate of 5 µL/min) by continuous infusion using the instrument syringe pump. The LCQ ESI source was operated at 4.5 kV, and the capillary heater was set to 200 °C, with nitrogen used as the nebulizing gas. For ESI-MS/MS experiments, the ions of interest were isolated monoisotopically in the ion trap and were activated by collisions. The helium damping gas that was present in the mass analyzer acted as a collision gas. The analysis was performed in the positive-ion mode. The low molar mass PHA oligomers resulting from the partial degradation of PHA using KHCO_3_ were purified from unreacted PS contamination by washing in water.

#### 2.6.6. PHA Molecular Analysis Procedure

PHA with low molar mass was acquired via the thermal degradation of bacterial PHA in the presence of potassium hydrogen carbonate (KHCO_3_) as described by Johnston et al. [5]. Samples were analyzed with the aid of GPC, FTIR, NMR spectrometry, and TGA, as well as oligomer structures at the molecular level using ESI-MS^n^ (Thermo Finnigan LCQ Fleet, Thermo Fisher Scientific Inc., San Jose, CA, USA).

## 3. Results

### 3.1. Acid Number of Carbon Sources (PS0–4)

The original prodegraded PS0 sample was found to have an acid number of 22 after titration; this was then compared to the treated samples (Table 1). To avoid the introduction of impurities in the fermentations, which may have a negative influence on bacterial growth, no initiators or catalysts were used in the PS treatment. Instead, ozone was applied to increase the rate of the oxidation, which could be performed with repeatability [5]. Using this process, the acid number of the PS samples could be controlled by the temperature. The hydrophobic nature of the PS flakes was, therefore, reduced under oxygen and ozone at 60–180 °C for up to 20 h and more hydrophilic groups were formed on the predominantly alkane chains of the prodegraded PS.

Table 1 shows the conditions of the oxo-degradation process and titrations with KOH confirmed acid numbers 23.6–39.0, showing the successful production of O–H, C=O groups. To assess the precise nature of the oxidized groups, FTIR was performed.

### 3.2. FTIR Analysis of Carbon Sources

**Figure 3:**
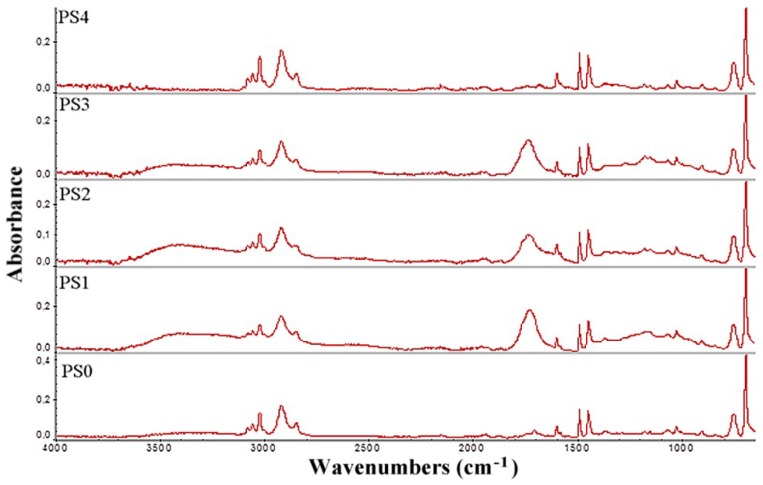
FTIR results of PS0–4 samples.

In all the spectra, the characteristic PS peaks (at 1452 and 1492 cm^−1^) correspond to C–H and –CH_2_ stretching bands that were similar to Yusof et al. [42]. Additionally, the broad peak of OH stretching (3400–3600 cm^−1^) and the peak absorptions in the regions of the carbonyl bands (1710–1730 cm^−1^) could be identified by FTIR spectroscopy. The region with maximum readings between 1710 to 1730 cm^−1^ (Figure 3) corresponds to the stretching vibrations of the C=O bonds originating from different functional groups of not only carboxylic acids, but also ketones or aldehydes, whose absorption bands may overlap [43].

What is worth noting is that after the thermal oxidation pretreatment process, the changes in the intensity of the OH and C=O bands for the samples tested were observed. The treatment of PS samples at 60, 80, and 100 °C show, in general, higher absorbance in the hydroxyl and carbonyl regions than the original material prior to oxidation. The PS samples after thermal oxidation at 180 °C present spectra with an absorbance profile similar to that recorded for PS0 before the pretreatment process. Therefore, PS1, PS2 and PS3 samples are better candidates for sources of activated carbon for selected bacteria strains. These results seem to be corroborated by the titration values presented earlier in Table 1.

### 3.3. GPC Analysis of Carbon Sources

The oxidation process of the PS specimens resulted in partial polymer degradation, and a reduction in the average molar mass, as shown in Table 2. In addition to adding ketone and aldehyde groups, the lower masses of the plastics increased the likelihood of bacterial metabolism. The dispersity (*Ð*) of the PS0–4 samples, as calculated from the *M*_w_/*M_n_* ratio, is also displayed in Table 2. There is little difference in *Ð* between all samples (2.8–3.0), apart from in PS4, which has a value of 3.8, indicating a more diverse number of polymer fragments within the sample, possibly formed by the prolonged heating and oxygen exposure conditions.

### 3.4. Thermal Analysis of Carbon Sources

The thermal decompositions of the investigated samples take place between 200 and 470 °C, when the gaseous phase is emitted. The PS0, PS1, PS2, PS3, and PS4 samples undergo one stage of mass loss. The horizontal step of the PS4 sample’s TG curve is narrower than that recorded for the other samples. This is confirmed by DTG curves in which PS0, PS1, PS2, and PS3 have visible bimodal peaks (Table 3).

The temperature of the maximum decomposition rate (*T*_max_) appears at around 411 °C. The content of inorganic material present in the samples can be determined based on the residue percentage (Table 3). The residue content increases with the increasing of the temperature during the pretreatment process.

### 3.5. NMR Analysis of Carbon Sources

**Figure 4:**
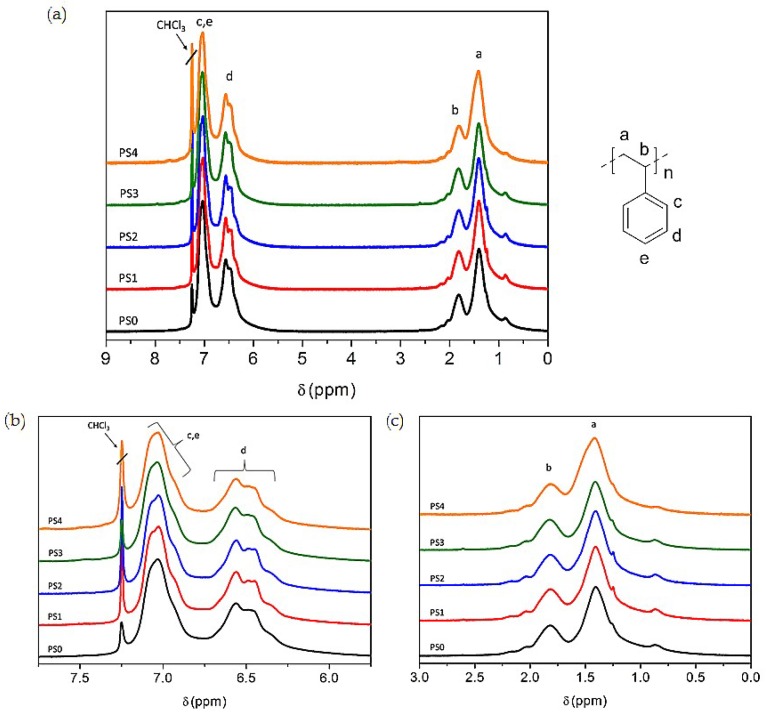
Stacked ^1^H-NMR spectra results of the PS0 (original) sample and PS1–4 (thermally treated) samples. Labels, a–e, represent the positions of hydrogen atoms as shown on the PS diagram featured. Graph (**a**) shows the overall spectra; (**b**) displays 7.5–6.0 ppm; (**c**) details 3.0–0.5 ppm.

The ^1^H-NMR results of the original PS0 sample are shown in Figure 4a–c and are consistent with documented PS spectra in the literature [44]. The strong signals observed between 6.0 and 7.3 ppm are attributed to pendant aromatic rings. The absence of any signals between 2.5 to 6.0 ppm indicates that no ester, alcohol, or halogen or vinyl functional groups are present. Signals at 1.4 and 1.8 ppm are the signals for the methylene and methane polymer backbone protons, respectively (assignments are shown in Figure 4a,c).

**Figure 5:**
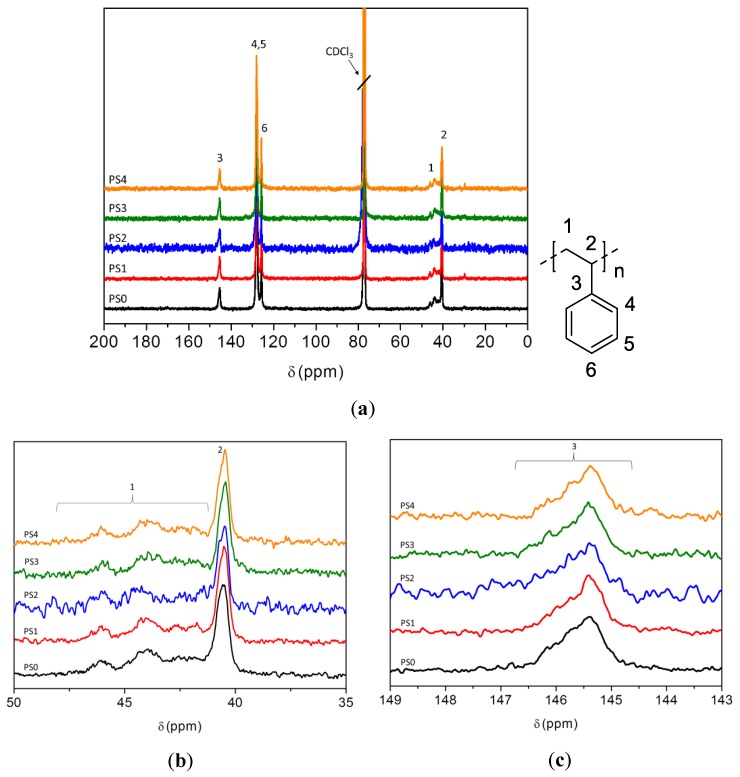
Full stacked proton decoupled ^13^C-NMR spectra of the PS0 (original) and PS1–4 (thermally treated) samples (**a**) and (**b**) expansions of the backbone methylene/methine (**c**) and *ipso* carbon regions ^1^H-NMR spectra of the PS1–4 (thermally treated) samples. Labels 1–6 indicate the carbon atoms on the PS diagram featured.

The ^13^C-NMR spectra (see Figure 5) show the carbon sources (PS0–4) are atactic polystyrene; this is clearly visible from inspection of both the methine (Figure 5b) and *ipso* (Figure 5c) carbon regions of the spectrum. Assignments of the carbon signals are displayed on the spectra and are in agreement with the assignments of London et al. [45] for the *ipso* region and Cheng and Lee [46] for the methylene region. The effect of thermo-oxidative degradation on the polymeric microstructure of the degraded samples (PS1–4) appears to be minor, with no major differences observable in either the proton or ^13^C spectra from that of PS0. No signals are observed in the carbonyl region of the ^13^C-NMR spectra of any of the samples (PS0–4). Additionally, there are no observable correlations within this region in the HMBC spectra. This is consistent with the relatively low acid number obtained from KOH titration (see Section 3.1).

### 3.6. Bacteria Growth

**Figure 6:**
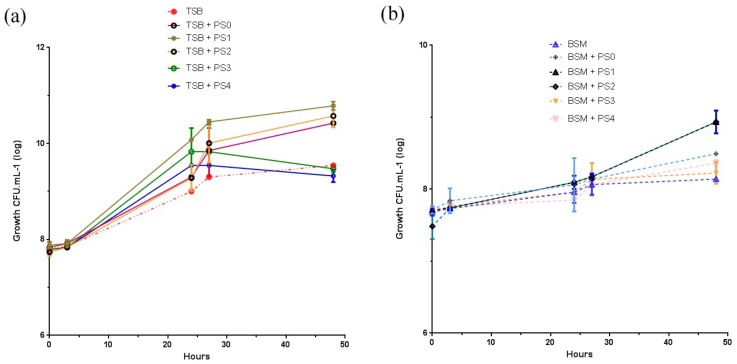
Fermentation of *C. necator* in growth media; each point is an arithmetic mean of triplicates, while error bars denote the standard error (SE) of the mean for comparison over 48 h. Graph (**a**) shows bacterial growth in TSB with PS0–4 as an additional carbon source and controls (**b**) shows growth in BSM, with PS0–4 as the only carbon source and controls.

The growth of *C. necator* in nitrogen rich TSB and in nitrogen low media (BSM) was conducted over a 48 h period with and without PS0–4 for comparison (Figure 6).

From a 25 mL starter flask of *C. necator* grown in TSB (48 h at 30 °C at 150 rpm), 1 mL of inoculum was pipetted into each conical flask with 250 mL of media (TSB/BSM) to ensure relatively close starting point numbers (log 10 CFU mL^−1^) of bacterial fermentations.

The nitrogen limited (BSM) fermentations did not yield PHAs and cell numbers were merely maintained with a maximum dry biomass of 0.16 g/L. The initial cell numbers of all cultures were 7.8 ± 0.2 log10 CFU mL^−1^. A two-way multiple ANOVA and Turkey’s multiple comparison confirmed there was no significance between BSM and BSM + PS0 CFU numbers (*P* = 0.37), indicating that PS0 alone is not enough for increased biomass.

With TSB (nitrogen rich medium) a maximum cell number of 10.0 ± 0.8 log10 CFU mL^−1^ was obtained (Figure 6a). This represents an increase in the log10 CFU mL^−1^ number of approximately 2.2 log10 CFU mL^−1^ across all nitrogen rich cultures. The results showed there was also no significance with PS4 and TSB (*P* = 0.76), and although the dry biomass recovered was 0.99 g, after further analysis, it was found that this sample contained low molecular mass (8000 Da) PS4 particles that passed through the extraction process, giving inaccurate results. This adjusted P value also suggests that PS4 was not metabolized and that the conditions (180 °C, O_3_/O_2_ for 20 h) used to produce PS4 (a powder rather than a flake) may have been too extreme to produce a viable carbon source, despite the average molecular mass being the lowest. The relatively dense, smaller PS4 particles were highly stable, crystalline, possessed low hydrophilic (AN = 23.6) properties, and were less structurally open as they are most likely in aromatic arrangements in comparison to the higher molecular mass PS flakes [47].

All other comparisons of PS0 and PS1–4 with TSB media against BSM fermentations showed significant results, including PS0 + TSB and TSB with a significance of *P* = 0.017, indicating that the addition of PS in either treated form increases cell numbers. Moreover, comparisons of PS1, PS2, and PS3 (the successfully oxidized prodegraded PS specimens) against BSM (Figure 6b) showed the greatest significance (*P* = 0.001) and they also yielded the highest dry biomass and PHA yields. The average cell dry weight (CDW) and the average PHA yield in grams per liter of fermentation media is presented in Table 4.

The cell growth analysis showed that prodegraded PS in TSB or BSM does not have a negative effect on the growth of *C. necator.* Previous experiments have shown that certain plastics, such as polyethylene (PE), have negligible antimicrobial properties against both gram positive and gram negative bacteria [35,48,49]. The low antimicrobial activity of some plastics could be due to the absence of antimicrobial moieties, such as chlorine or fluorine, within the polymer chains, which could be released during an oxidation process. They could then leach out into the surrounding media [50]. However, PS is considered relatively stable and it is widely thought of as a safe plastic for food and drink packaging.

### 3.7. PHA Identification and Characterization

#### 3.7.1. FTIR Results

**Figure 7:**
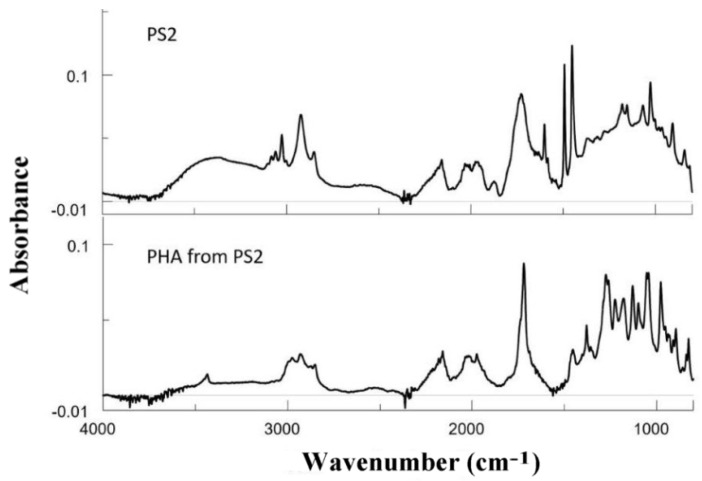
The comparison of the FTIR spectra of PS2 with the spectra of PHA produced from PS2 after 48 h fermentation.

From the spectra of PHA samples in Figure 7, it can be seen that the absorbance of peaks characteristic of PS at 1452 and 1492 cm^−1^ are smaller compared with the carbonyl region in the same sample. For PS samples, the peaks at this and at the carbonyl regions have comparative absorbance. Additionally, in the FTIR spectra of the PHA samples, in the region of 600–1500 cm^−1^, the peaks correspond to C–C aliphatic bonds of PHA that were documented by Shamala et al. [51].

#### 3.7.2. PHA GPC Results

**Figure 8:**
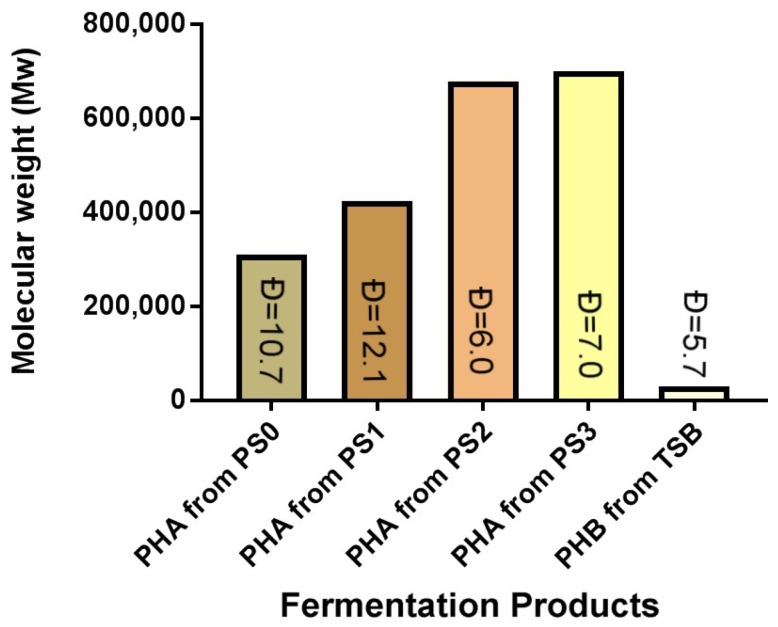
The GPC results with dispersity (*Ð*) from PHAs produced. PHB from TSB (control) and PS0–3 samples as additional carbon sources with TSB (nitrogen rich) media. No PHAs were produced in BSM only or BSM with PS0–4 fermentations or any TSB with PS4 only fermentations.

The results of molar mass measurements of the biopolymers synthesized by the bacterial fermentations were estimated by GPC, using polystyrene standards. The results are shown in Figure 8. No PHAs were produced in the BSM media with PS0–4 or in the control flasks without additional carbon sources. When PS4 was used as a carbon source, the fine particles of the PS powder contaminated the dry biomass and no PHA was produced. PHAs from PS1 showed the greatest dispersity (*Ð* = 12.1).

#### 3.7.3. Thermal Analysis of PHA

The thermal decomposition of PHA produced from PS0, PHA from PS1, and PHA from PS2 samples underwent two stages of mass loss. This means that the samples tested contained not only PHAs, but also PS fragments that passed through post-fermentation chemical processing. Thus, the second step of the process corresponded to the PS sample’s component thermal decompositions. The percentages of content of PSx can be calculated from the residue after the first step (Table 5).

#### 3.7.4. NMR and ESI-MS Results

**Figure 9:**
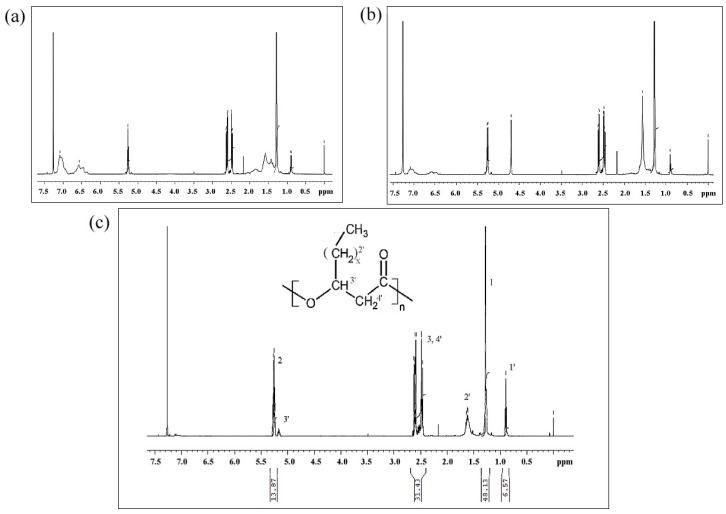
^1^H-NMR of PHAs produced from (**a**) PS0; (**b**) PS1; and (**c**) PS2 as an additional carbon source in TSB media.

The ^1^H-NMR of PHAs produced from the samples, PS0, PS1, and PS2, as an additional carbon source in TSB media are shown in Figure 9.

The ^1^H-NMR spectra of PSx-PHA revealed the presence of characteristic signals corresponding to the protons of 3-hydroxybutyrate (3-HB) and 3-hydroxyvalerate (3-HV) repeating units. From the integration of the signals of methyl groups derived from 3-HB (1.28 ppm) and 3-HV (0.9 ppm), the chemical compositions of PHA obtained were calculated and are presented in Table 6. Unfortunately, on the NMR spectra, there are broad signals at 1.5, 1.8, and 7.1 ppm showing likely contamination from PS (see Figure 9c); these results are consistent with the previous TGA data. Moreover, the ^1^H-NMR results observed for the PHA produced from PS2 samples present a smaller amount of contamination from PS, which is also consistent with the previously reported TGA results.

Table 6 also illustrates that sample composition change was not due to thermal degradation, and any differences in HV unit content might be due to the bacterial synthesis. ^13^C-NMR was also performed, which corroborated these ^1^H-NMR results, but offered no additional information (data not shown).

**Figure 10:**
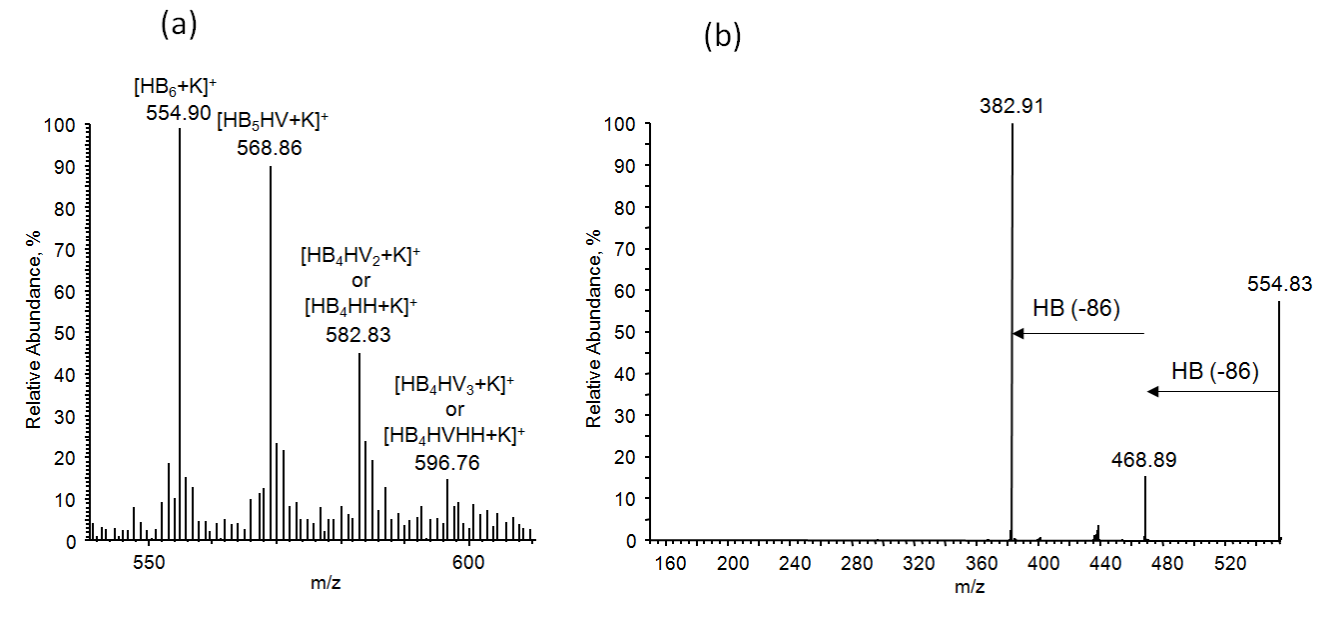
(**a**) The expanded spectral range at *m/z* 500 to 650 of the ESI-MS spectrum of low molar mass PHA oligomer; (**b**) the ESI-MS/MS spectrum of selected potassium adduct ions of oligomers [HB_6_ + K]^+^ at *m/z* 555.

The results from the partial degradation of the PHA low molar mass PHA oligomers (below 2000 Da) needed for ESI-MS/MS analysis were found to possess nearly the same content of HB units as revealed by ^1^H-NMR (Table 6). The expanded region of the ESI-MS spectrum of the low molar mass PHA oligomer (resulting from the partial degradation of PHA obtained using PS2 as a carbon source) acquired in the positive ion mode is presented in Figure 10a. The most intense ions of the analyzed cluster were potassium adduct ions at *m/z* 555, 569, 583, and 597.

**Scheme 1:**
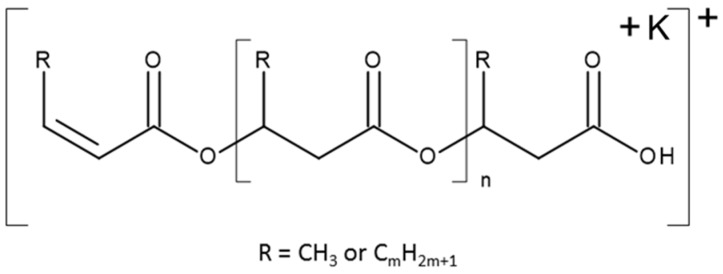
General formula of ions observed in the ESI-MS spectra.

The expanded spectral range at *m/z* 500 to 650 indicates the presence of ions with a mass difference between the *m/z* values equal to 14 Da (i.e., *m/z* 555, 569, 583, and 597), which corresponds to the difference between the molar mass of the individual co-monomeric units with longer side chains. The general structure of the ions presented in the ESI-MS spectrum is shown in Scheme 1.

The ESI-MS/MS fragmentation experiments were conducted for selected cluster ions. The ESI-MS/MS product ions spectrum for the selected potassium adduct of the oligomer, [HB_6_ + K]^+^, at *m/z* 555 is presented in Figure 10b. The fragmentation of this ion, which was obtained from the random breakage of ester bonds along both sides of the oligomer chain, led to the formation of only one set of oligo(3-hydroxybutyrate) product ions at *m/z* 469 and 383, terminated by crotonate and carboxyl end groups. The first fragment ion from this series at *m/z* 469 was formed by the displacement of a neutral molecule of crotonic acid (86 Da) from both ends of the parent ion at *m/z* 555. The fragmentation spectrum of this ion confirms that the most intensive ions in the clusters correspond to potassium adducts of 3-hydroxybutyrate oligomers.

**Figure 11:**
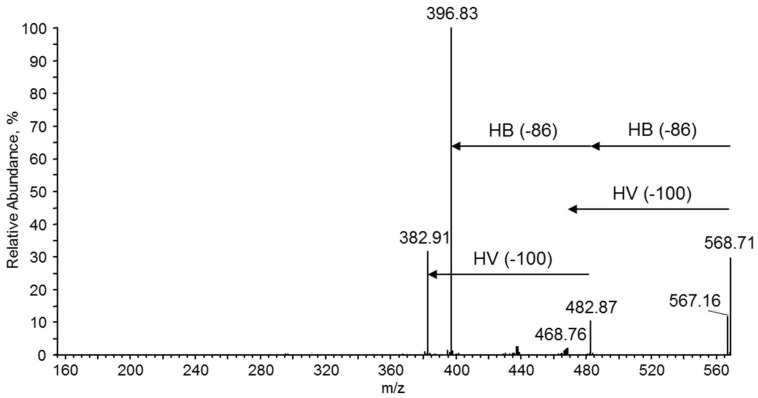
ESI-MS/MS spectrum obtained for the selected potassium adduct ion of the oligomers, [HB_5_ HV + K]^+^, at *m/z* 569.

In Figure 11, the ESI-MS/MS spectrum of the selected parent ion, [HB_5_ HV + K]^+^, at *m/z* 569, which contains 5 HB and one HV unit, is presented.

**Figure 12:**
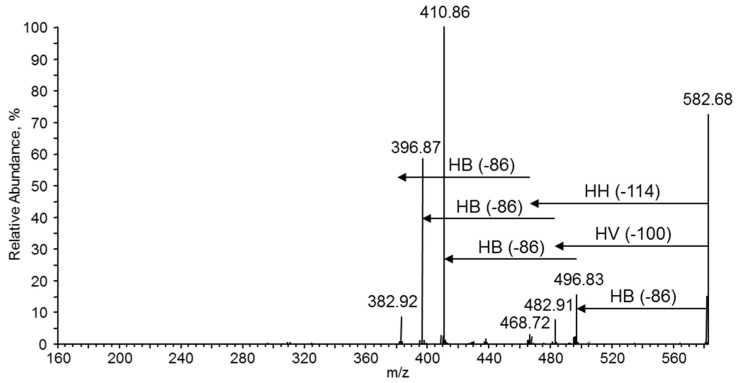
ESI-MS/MS spectrum acquired for the selected potassium adduct of oligomers at *m/z* 583.

However, Figure 12 presents the three fragmentation paths observed for the parent ion *m/z* 583 that will be discussed in Section 4.

**Scheme 2:**
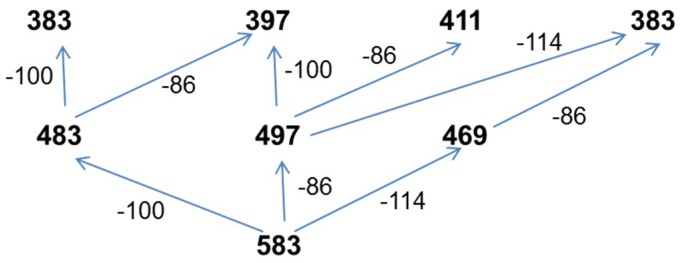
Proposed fragmentation paths of the parent ion *m/z* 583 observed in the ESI-MS/MS spectrum.

Scheme 2 presents the proposed fragmentation paths for the parent ion of *m/z* 583 shown in Figure 12.

## 4. Discussion

The direct recycling of postconsumer plastics, such as PS, would lead to products of poor quality, with a loss of properties due to aging during the service-life and any thermo-mechanical degradation during reprocessing [52]. By using prodegraded PS as a carbon source to produce biodegradable plastics, waste PS gains an extended usefulness time-span, rather than becoming a hazardous material. The results of Table 1 and the FTIR data (Figure 3) clearly show that the thermal pretreatment with ozone increased the presence of ketone or aldehyde groups, further increasing the acid number of samples PS1–3. A treatment time of 20 h at 60 °C was even more effective than the treatment at 80–100 °C for the same period and when 120 °C was used for 20 h the samples are rendered useless for bacterial fermentation.

The GPC results showed a progressive decrease in the average molecular mass of the PS samples when heat exposure was extended. This was expected, however, it was not known that PS4 samples, although possessing the lowest molecular mass, would produce the least biomass and no PHAs after 48 h fermentation. The dispersity (*Ð*) between the samples was mostly consistent, apart from PS4 (*Ð* = 3.8), possibly showing a higher range of polymers in the sample that could have been produced by the thermal treatment itself. As there was very little growth and no product, these particles could be hazardous so further analysis, or simply avoiding the more extreme conditions used to produce the PS4 samples, is suggested if this kind of pretreatment is utilized by laboratories in the future. It is known that the presence of mixed polymeric fractions, such as impurities in recycled polymeric materials, can affect the macroscopic performance of recyclates [23]. By using thermal analysis as part of the characterization analysis, it has been observed that the PS fragments were able to pass through the extraction into the PHA because PS readily dissolves in chloroform. If this process was to be adopted in an industrial setting, further filtration steps would have to be added.

From these results in Section 3.6, it does appear that the treated PS samples significantly increased the biomass and percentage yield of the PHA. Comparisons of PS1, PS2, and PS3 (the successfully oxidized PS specimens) against BSM showed the greatest significance (*P* = 0.001), with PS1 showing a 48% yield. The combination of increased hydrophilicity and reduced average molecular mass undoubtedly contributed to the better emulsification and absorption of the PS carbon chains by the bacteria into the β*-*oxidation pathway, producing PHBV rather than PHB as in the TSB only controls. PS1 sample fermentations also produced relatively low average molecular mass PHAs of 35,000 Da, with the highest distribution value of 12.1 (*M*_w_/*M*_n_), compared to PS2 (113,000 Da with *Ð* = 6.0) and PS3 (100,000 Da with *Ð* = 7.0). When these data are compared to the *M*_n_ results for polyesters synthesized using TSB only, the PHAs synthesized showed a *M*_n_ of 52,000 Da with a distribution of 5.7 (*M*_w_/*M*_n_) [5]. This shows that PS1 samples had the greatest dispersity value, and more varied ranges of polyesters were generated in all prodegraded sample fermentations, but PS1 was the only pretreated sample to give a dispersity value greater than the original prodegraded (PS0) specimen. Again, this is likely due to the higher AN value.

^1^H-NMR was used to determine the molecular structure of the copolymers produced using fermentation. The ^1^H-NMR spectra of PSx-PHA revealed the presence of characteristic signals corresponding to the protons of 3-hydroxybutyrate (3-HB) and 3-hydroxyvalerate (3-HV) repeating units. It was found that PHB was produced by bacteria in the TSB only fermentations. After integrating these signals, the content of monomer units other than 3-hydroxybutyrate (3-HB) has been estimated at 12 mol % 3-HV.

ESI-MS determined the molecular structure of the copolymers that were included in the PHAs produced from the prodegraded PS waste samples. This method is known to provide more detail than ^1^H-NMR or GC when it is applied for PHA characterization. This is because it can corroborate whether a copolymer is randomly distributed or a block copolymer [53]. Therefore, it enables the precise sequence analysis of oligomer distribution using the fragmentation technique. The ESI-MS/MS product ions’ spectrum for the selected potassium adducts of the oligomer, [HB_6_ + K]^+^, at *m/z* 555 shown in Figure 10b confirms that the most intensive ions in the clusters correspond to potassium adducts of 3-hydroxybutyrate oligomers. In Figure 11, the ESI-MS/MS spectrum for the selected ion, [HB_5_ HV + K]^+^, at *m/z* 569 is presented. During the fragmentation, the breakage of ester bonds along the oligomer chains led to the formation of two series of product ions at *m/z* 483, 397 and at *m/z* 469, 383. Thus, the product ion at *m/z* 483 corresponded to the oligomer formed by the loss of crotonic acid (86 Da) while the product ion at *m/z* 469 was formed due to the loss of a valeric acid (100 Da) unit, as seen from the fragmentation pathway shown in Figure 11. Therefore, the fragmentation spectrum acquired for the precursor ion at *m/z* 569 confirms the presence of 3-hydroxybutyrate and the 3-hydroxyvalerate co-monomer units randomly distributed along the polyester chains.

The ion at *m/z* 583, presented in Figure 10a, may corresponded to the isobaric potassium adduct ions containing two HV units or one HH unit [HB_5_ HV_2_ + K]^+^ or [HB_5_ HH +K]^+^, respectively. Conversely, three fragmentation paths were observed in the case of the ESI-MS/MS spectrum of the parent ion at *m/z* 583 (Figure 12). Two series at *m/z* 496, 411 and at *m/z* 483, 383 were formed in the same way as described for fragmentation of the ion at *m/z* 569 (Figure 11). However, the third series at *m/z* 467, 383 indicates that the first ion in this series is formed by the expulsion of 2-hexenoic acid (114 Da), which unequivocally confirms the presence of 3-hydroxyhexanoate (3-HH) units in the oligomer chain. Thus, the fragmentation experiment performed for the ion at *m/z* 583 indicates the presence of three co-monomeric units in this oligomer; namely, 3-hydroxybutyrate and 3-hydroxyvalerate as well as 3-HH co-monomer units, which are randomly distributed along the oligomer chain. It is interesting to note that the fragmentation results are similar to those reported previously in the presence of oxidized polyethylene waxes as carbon sources for bacteria to produce PHAs, as described by Radecka et al. [35].

The significance of the ratio of PHB to PHBV is that properties, such as melting point or mechanical strength, are changed; therefore, the applications of the bioplastic synthesized could be determined. In the case of PHAs, these applications include: General packaging of differing biodegradation rates, electrospinning materials, and 3D printing filaments [7,54]. PHAs that contain functional groups are also extremely appealing for biomedical usage as they allow for further chemical modification. This could be in the form of bioactive oligomers that distribute drugs using pH-controlled release [55]. Polyethylene glycol (PEG) is a polyether that has been combined with PHA to alter its properties. Bulk polymerized PHBHHx/PEG poly (ether ester urethane) block copolymers have shown improved tissue compatibility, with greater physicochemical properties when used in vivo as biofilms [56,57]*.* In addition, recombinant *E. coli* enzymatic systems combined with fermentation optimization for PHA functionalization have been used to synthesize PEGylated PHB-co-PHHx copolymers with improved tissue regeneration abilities in comparison to PHB-co-PHHx and PHB, as well as “clickable” unsaturated PHA chains. Consequently, the PHA-polymer components for all these applications could originate from waste PS [57,58].

## 5. Conclusions

To assess the quality of the recycled GPPS and HIPS, an integrated approach was applied, involving different analytical strategies, such as GPC, FTIR, NMR, and ESI-MS/MS. From these analytical techniques, the chemical structure and morphological properties of the prodegraded PS0 and pretreated PS1–4 materials were noted. We have demonstrated that PHAs could be synthesized using PS0–3 samples as a carbon source. Even though there was a degree of mixing contamination of low molecular weight compounds from the PS samples used in the fermentation experiments of this study, to produce PHAs, waste prodegraded PS has proven to be a viable raw material. In fact, the contamination could alter the mechanical properties of the PHA synthesized, offering alternative applications.

We propose that the difference in samples may be evidence of the emission of volatiles during processing, and the formation of degradation products in other cases. The PS1 sample was the most energy efficient treated carbon source, with the most hydrophilic structure and produced the most biomass, with a promising 48% PHA yield. As such, unwanted PS could potentially join the group of carbon-rich industrial waste streams (“2nd generation feedstocks”) that can be used as an alternative to carbon sources that are derived from food and feed supplies. This method would not only reduce PHA production costs, and contribute towards safeguarding food and feed supplies in various disadvantaged parts of the world, but also help reduce the PS content in landfills. The aims of the EU, within the vision of a circular economy, are to reduce the leakage of plastics into the environment and recycle, which could be assisted by using oxo-degradation to form carbon sources for industrial fermentation. This proposed strategy could be employed alongside other sustainable chemical techniques to tackle sea-based marine litter particularly, including PS, GPPS, HIPS, and other types of plastic packaging.

## 6. Patents

Partial degradation of PHA is a subject of EU patent entitled: PROCESS FOR CONTROLLED DEGRADATION OF POLYHYDROXYALKANOATES AND PRODUCTS OBTAINABLE THERE FROM. EP 2 346 922 B1. International publication number: WO 2010/044112 (22.04.2010 Gazette 2010/16). Date of filing: 15.10.2008. Date of publication of application: 27.07.2011.

## Figures and Tables

**Table 1:** Conditions and acid number results of thermal degradation procedure on PS0 flakes.

Sample	Acid Number (mg KOH/g)	Process Conditions
PS0	22.0	N/A
PS1	39.0	60 °C, O_3_/O_2_, 20 h
PS2	33.7	80 °C, O_3_/O_2_, 20 h
PS3	29.5	100 °C, O_3_/O_2_, 20 h
PS4	23.6	180 °C, O_3_/O_2_, 20 h

**Table 2:** The average molar mass (*M*_n_), average molecular weight (*M*_w_*),* and dispersity (*Ð*) of the PS0–4 samples.

Sample	*M*_w_	*M*_n_	*Ð* (*M*_w_/*M*_n_)
PS0	65,000	22,000	3.0
PS1	57,000	19,000	3.0
PS2	48,000	17,000	2.8
PS3	42,000	14,000	3.0
PS4	30,000	8000	3.8

**Table 3:** The Thermogravimetric parameters for PS1–4 samples.

Sample Name	*T*_max_ (°C)	Residue (%)
PS0	408/443	2.1
PS1	411/441	3.9
PS2	411/441	4.2
PS3	411/439	4.3
PS4	411	4.6

**Table 4:** The amount of PHA synthesized by *C. necator* using 4 g/L PS conducted over a 48 h incubation period at 30 °C (150 rpm). ND = non-detected; CDW = cellular dry weight.

Media	Average CDW (g/L)	Average PHA (g/L)	PHA (% *w*/*w*)
BSM only	0.08 ± 0.06	ND	ND
TSB only	1.61 ± 0.60	0.38 ± 0.03	17%
PS0 + BSM	0.08 ± 0.02	ND	ND
PS0 + TSB	1.33 ± 0.20	0.52 ± 0.05	39%
PS1 + TSB	3.56 ± 0.02	1.72 ± 0.03	48%
PS2 + TSB	3.04 ± 0.03	1.28 ± 0.02	42%
PS3 + TSB	2.64 ± 0.04	0.96 ± 0.03	36%
PS4 + TSB	3.96 * ± 0.05	ND	ND

* Dry biomass of PS4 experiments contained PS4 particles that could not be separated using filtration.

**Table 5:** The thermogravimetric parameters for the PHA samples produced after 48 h fermentation with PS0–2 as an additional carbon source in TSB media.

Sample Name	*T*_max_ (°C)	Residue after First Step (%)	Residue after Second Step (%) (Inorganic Part)
PHA from PS0	295/435	51.8	2.3
PHA from PS1	294/435	27.0	1.5
PHA from PS2	289/435	10.0	1.7

**Table 6:** The chemical composition of PSx-PHA samples before (0) and after partial depolymerization for ESI-MS/MS analysis (D) (determined from ^1^H-NMR analysis).

Sample Name	Sample Composition
	Content of Non-HB Units (%)
PS1-PHA0	8.2
PS1-PHAD	8.3
PS2-PHA0	12.1
PS2-PHAD	11.5
